# Increased Incidence of Tinnitus Following a Hyperthyroidism Diagnosis: A Population-Based Longitudinal Study

**DOI:** 10.3389/fendo.2021.741719

**Published:** 2021-11-03

**Authors:** Tang-Chuan Wang, Chien-Jen Chiu, Pei-Chun Chen, Ta-Yuan Chang, Richard S. Tyler, Eveling Rojas-Roncancio, Claudia Barros Coelho, Patricia C. Mancini, Cheng-Li Lin, Chia-Der Lin, Ming-Hsui Tsai

**Affiliations:** ^1^Department of Public Health, College of Public Health, China Medical University, Taichung, Taiwan; ^2^School of Medicine, College of Medicine, China Medical University, Taichung, Taiwan; ^3^Department of Otolaryngology-Head and Neck Surgery, China Medical University Hsinchu Hospital, Hsinchu, Taiwan; ^4^Department of Public Health, College of Public Health, China Medical University, Taichung, Taiwan; ^5^Department of Occupational Safety and Health, College of Public Health, China Medical University, Taichung, Taiwan; ^6^Department of Otolaryngology-Head and Neck Surgery, University of Iowa, Iowa City, IA, United States; ^7^Otorrinolaringóloga, Universidad Nacional-Universidad Militar, Miembro Asociación Colombiana de Otorrinolaringología, Cirugía de Cabeza y Cuello, Maxilofacial y Estética Facial (ACORL), Bogotá, Colombia; ^8^College of Medicine, University of Vale do Taquari (UNIVATES), Center of Medical Sciences, Rio Grande do Sul, Brazil; ^9^Department of Speech-Language Pathology and Audiology, Universidade Federal de Minas Gerais, Belo Horizonte, Brazil; ^10^Management Office for Health Data (DryLab), Clinical Trial Center (CTC), China Medical University Hospital, Taichung, Taiwan

**Keywords:** hyperthyroidism, incidence, risk factors, tinnitus, cohort study, national health insurance research database

## Abstract

**Background:**

An association between thyroid disease and tinnitus has been described previously but further longitudinal, population-based studies are limited.

**Objective:**

To investigate the incidence of tinnitus in patients with hyperthyroidism in a national sample, and to identify risk level and associated factors for tinnitus in hyperthyroidism patients.

**Design:**

Retrospective cohort study. Patient data were collected from the Longitudinal Health Insurance Database (LHID 2000), which includes national claims data of patient expenditures for admissions or ambulatory care from 1996 to 2011.

**Setting:**

Taiwan hospitals and clinics providing healthcare nationwide.

**Participants:**

Patients aged 20 years and older with newly diagnosed hyperthyroidism (ICD-9-CM code 242) between 2000-2010 were selected as the study cohort. Hyperthyroidism patient cohort were identified from the LHID2000. Those with tinnitus history (ICD-9-CM code 388.3) before the index date (first hyperthyroidism diagnosis), younger than 20 years, and with incomplete demographic data were excluded. The non-hyperthyroidism cohort included patients with no history of hyperthyroidism and no documented tinnitus.

**Main Outcomes and Measures:**

Incidence of tinnitus was the primary outcome. Baseline demographic factors and comorbidities possibly associated with tinnitus, including age, sex, and comorbidities of hearing loss, vertigo, insomnia and anxiety, were retrieved from the LHID 2000. Patients were followed until end of 2011.

**Results:**

During the study period, 780 (4.9%) hyperthyroidism patients and 2007 (3.2%) non-hyperthyroidism controls developed tinnitus. Incidence rate of tinnitus in the hyperthyroidism cohort was significantly higher in hyperthyroidism cohort (7.86 *vs.* 5.05 per 1000 person-years) than that in non-hyperthyroidism cohort. A higher proportion of patients with hyperthyroidism had comorbid insomnia (45.1% *vs.* 30.9%) and anxiety (14.0% *vs.* 5.73%) than those without hyperthyroidism. After adjusting for age, gender and comorbidities (vertigo, insomnia, anxiety, hearing loss), hyperthyroidism patients had 1.38-fold higher risk of tinnitus (95% CI = 1.27-1.50) than those without hyperthyroidism.

**Conclusions:**

This large population-based study suggests patients with diagnosed hyperthyroidism was more prone to develop tinnitus. Our findings suggest evaluation for comorbid vertigo, insomnia, anxiety and/or hearing loss may identify patients who are at high risk of developing tinnitus in patients with hyperthyroidism.

## Introduction

Tinnitus refers to the perception of hearing sounds with no identifiable external acoustic source. Affected individuals self-report that tinnitus disturbs thoughts and emotions, interferes with hearing, and interrupts sleep and concentration, although many still consider it to be harmless (1, 2). A cross-sectional study using data from the US National Health and Nutrition Survey (NHANES) found that prevalence of frequent tinnitus was highest (14.3%) among older adults and the odds of frequent tinnitus increased when associated with low-mid and high frequency hearing loss, loud noise exposure, smoking, hypertension and general anxiety disorder (3). However, although tinnitus is prevalent in both genders and all ages, its true global prevalence is not known. A recent systematic review of global tinnitus-related studies from 1980 to 2015 emphasized the difficulty in finding studies with consistent definitions of tinnitus, particularly given the absence of standard diagnostic criteria or methods, and that tinnitus is typically self-reported and may go unrecorded (4). Prevalence among 515 studies ranged widely from 5.1%-42.7%, with agreement between studies shown only for prevalence increasing with age.

Previous studies have reported associations between tinnitus and aging, gender, noise exposure, insomnia, bilateral hearing loss and various diseases and syndromes, including cardiovascular disease, epilepsy, anxiety syndrome and others (5–8). Among patients with chronic tinnitus, the presence of lifetime and current psychiatric disorders was 60% and 55%, respectively, and the majority were depressive and anxiety disorders (9). Frequent tinnitus was also associated with anxiety syndrome in patients undergoing mental health evaluation (3). A population-based study examining risk factors associated with tinnitus found increased odds of developing tinnitus in those with history of hyperlipidemia, osteoarthritis, rheumatoid arthritis and thyroid disease (2). In that cross-sectional study, a history of thyroid disease was significantly associated with tinnitus (OR 1.59).

Tinnitus has been found in patients with hyperthyroidism, hypothyroidism and autoimmune thyroiditis (10). However, although the association between hyperthyroidism and tinnitus has been reported previously in cross-sectional studies, in-depth studies of the causal-relationship are lacking, particularly longitudinal cohort population-based studies, which may yield more definitive results for prevalence of tinnitus as well as confirming the association. We believe that the association between hyperthyroidism and tinnitus has been understudied, and more attention is deserved. This study aimed to investigate the incidence of tinnitus in a national sample of hyperthyroidism patients and to identify level of risk and associated factors for tinnitus in patients with hyperthyroidism.

## Methods

### Data Source

All data for this retrospective cohort study were retrieved from the Taiwan Longitudinal Health Insurance Database 2000 (LHID2000), which is a subset data of the National Health Insurance Research Institute Database (NHIRD) in Taiwan. LHID2000 included one million representative beneficiaries randomly selected from the NHIRD database in 2000 and contains data from 1996 to 2011. The National Health Insurance (NHI) program in Taiwan is a universal insurance system established in 1995 and implemented by the Bureau of National Health Insurance of the Taiwan Department of Health, and the program now covers about 99% of the Taiwan population (23.74 million) (http://www.nhi.gov.tw). The NHIRD is a population-level data source for real-world evidence to support clinical decisions and healthcare policy (11).

Confidentiality of beneficiaries is maintained according to the directives of the Bureau of NHI. Diseases in the LHID2000 are identified based on the International Classification of Diseases, 9th Revision (ICD-9). The LHID2000 provides encrypted patient personal information for the protection of privacy and provides researchers with anonymous identification numbers that connect to the relevant claim information, including patients’ sex, date of birth, medical services received and drug prescriptions.

### Ethics Statement

The protocol for this study was approved by the Institutional Review Board of China Medical University in central Taiwan (CMU-REC-101-012). Because all patient data in the LHID are deidentified, signed informed consent was waived for this study.

### Study Population

The data of newly diagnosed hyperthyroidism patients (ICD-9-CM code 242) between 2000 to 2010 were retrieved. The index date was defined as the date of diagnosis of hyperthyroidism. Those with tinnitus history (ICD-9-CM code 388.3) before the index date, aged less than 20 years, and with incomplete information on demographics were excluded. A comparison cohort was selected from LHID 2000 patients with no history of hyperthyroidism, and no tinnitus documented. The hyperthyroidism cohort and non-hyperthyroidism comparison cohort were frequency-matched in a 4:1 ratio according to age (every 5-years stratum), sex, and index-date. Finally, the data of a total of 15874 patients with hyperthyroidism (hyperthyroidism cohort) and 63496 patients without hyperthyroidism (non-hyperthyroidism cohort) were included as the analytic sample in this study.

### Main Outcomes and Study Variables

All included patients were followed to investigate the risk of developing tinnitus during the follow up period, from the index date to tinnitus diagnosis, withdrawing from the program (death), or at the end of 2011, whichever came first. Comorbidities were defined before the index date. Comorbidities in this study were defined by ICD-9 codes from inpatient or outpatient claims from the NHI, including vertigo (ICD-9-CM code 386), insomnia (ICD-9-CM code 780), anxiety (ICD-9-CM code 300.00), and hearing loss (ICD-9-CM codes 388-389).

### Statistical Analysis

Differences between the two cohorts in the distribution of categorical variables such as age, sex, urbanization level and comorbidities were determined using the Chi-square test. Differences in continuous variables between the two cohorts were determined using the *t*-test. Overall age, gender, comorbidities and follow-up years for tinnitus (per 1000 person-years) were calculated for the two cohorts. Univariable and multivariable Cox proportional hazard regression analysis was performed to evaluate the association between hyperthyroidism and tinnitus and the risk of developing tinnitus in hyperthyroid patients, expressed as hazard ratios (HR) and 95% confidence intervals (CI). A multivariable model was used to adjust for age, sex and comorbidities of vertigo, insomnia, anxiety, and hearing loss. The cumulative incidence for tinnitus between the two cohorts was plotted by Kaplan-Meier analysis and the differences were tested by Log-rank test. All statistical analysis was performed using SAS 9.3 statistical software (SAS Institute Inc., Cary, NC, USA). The significance level was set as p <0.05 (two-tailed).

## Results

### Baseline Demographic and Clinical Characteristics

Baseline characteristics of patients in the two cohorts—with and without hyperthyroidism—are presented in **Table 1**. Women (77.6%) and patients aged >40 years (53.0%) represented the major proportion of both cohorts. The hyperthyroidism cohort had a significantly higher mean age than those in the non-hyperthyroidism cohort (43.2 ± 14.6 *vs.* 42.9 ± 15 yo, respectively). The distribution of urbanization levels was significantly different between the two cohorts, and a higher proportion of high-urbanization levels (level 1 and level 2) was found in the hyperthyroidism cohort than in the non-hyperthyroidism cohort. Compared to subjects without hyperthyroidism, patients with hyperthyroidism were significantly more likely to have vertigo (7.40% *vs.* 4.65%), insomnia (45.1% *vs.* 31.2%), anxiety (14.0% *vs.* 5.83%), and hearing loss (1.22% *vs.* 0.81%) (all p values <0.001).

**Table 1:** Baseline characteristics of patients with and without hyperthyroidism.

	Hyperthyroidism		*p*-value
	No	Yes	
	(N = 63496)	(N = 15874)	
Gender			0.99
Women	49260 (77.6%)	12315 (77.6%)	
Men	14236 (22.4%)	3559 (22.4%)	
Age stratified			0.99
≤40	29852 (47.0%)	7464 (47.0%)	
>40	33644 (53.0%)	8410 (53.0%)	
Age, mean ± SD^a^	42.9 ± 15.0	43.2 ± 14.6	0.03
Urbanization level			<0.001
1 (highest)	20198 (31.8%)	5252 (33.1%)	
2	18716 (29.5%)	4717 (29.7%)	
3	11397 (18.0%)	2715 (17.1%)	
4 (lowest)	13185 (20.8%)	3190 (20.1%)	
Comorbidity			
Vertigo	3029 (4.77%)	1175 (7.40%)	<0.001
Insomnia	19648 (30.9%)	7162 (45.1%)	<0.001
Anxiety	3639 (5.73%)	2214 (14.0%)	<0.001
Hearing loss	461 (0.73%)	193 (1.22%)	<0.001

Chi-Square Test, ^a^t-test.

^†^The urbanization level was categorized by the population density of the residential area into 4 levels, with level 1 as the most urbanized and level 4 as the least urbanized.

### Incidence of Tinnitus Between Patients With and Without Hyperthyroidism

**Figure 1:**
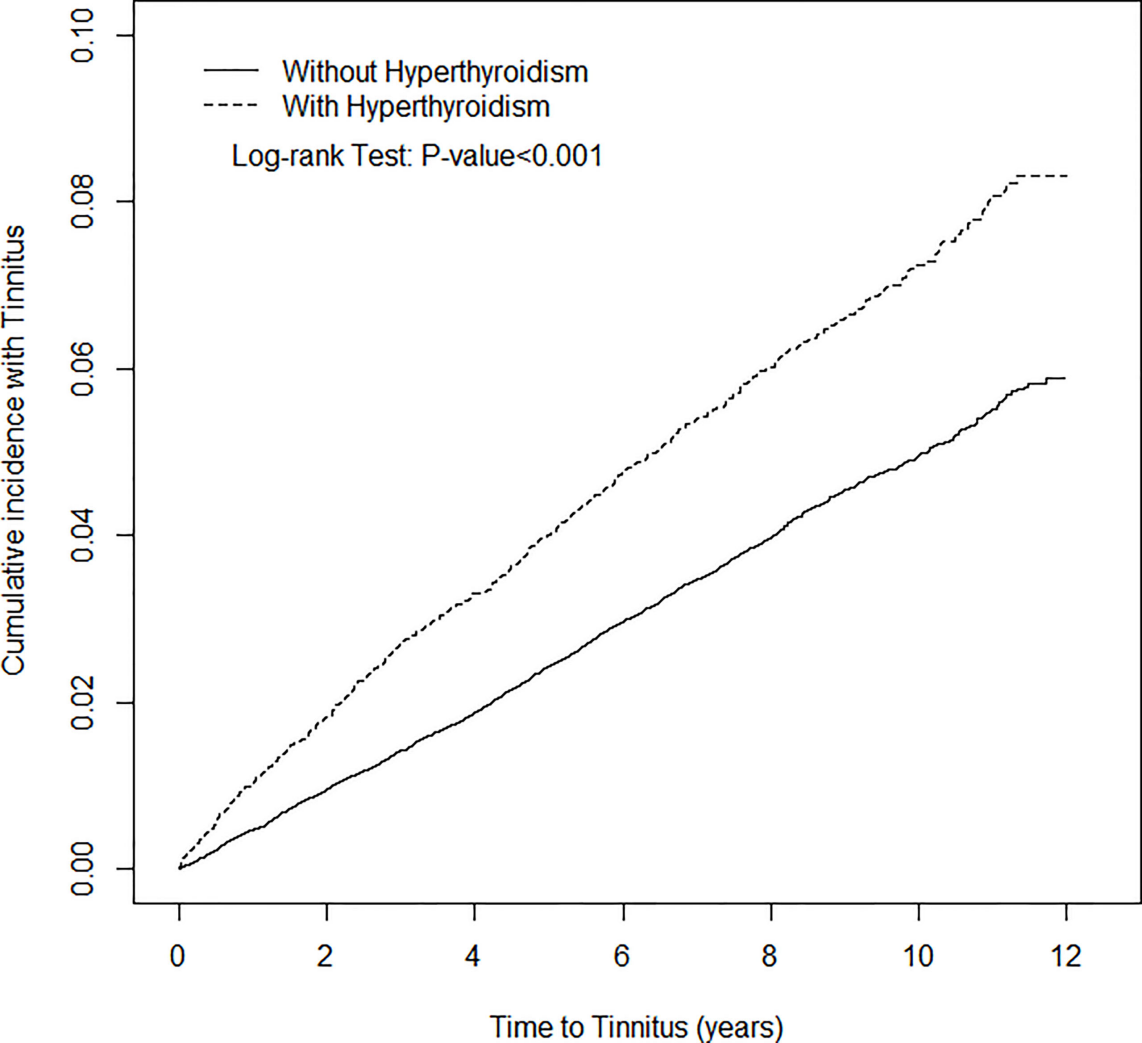
Cumulative incidence of Tinnitus for patients with (dotted line) or without (solid line) Hyperthyroidism.

After 12 years of follow-up, the cumulative incidence of tinnitus in the hyperthyroidism cohort was approximately 2.3% higher than that in the non- hyperthyroidism cohort (Log-rank p<0.001) (**Figure 1**). The mean follow-up time was 6.25 (SD=3.51) years and 6.26 (SD=3.50) years for the hyperthyroidism cohort and non-hyperthyroidism cohort, respectively. During the study period, 780 (4.9%) hyperthyroidism patients and 2007 (3.2%) non-hyperthyroidism controls developed tinnitus. The incidence of tinnitus in hyperthyroidism patients was 1.56-fold greater (7.86 *vs.* 5.05 per 1000 person-years) than that in non-hyperthyroidism controls (**Table 2**).

**Table 2:** Hazard ratios of tinnitus incidence between patients with and without hyperthyroidism by demographic characteristics and comorbidities.

	Hyperthyroidism						Crude HR* (95% CI)	Adjusted HR^†^ (95% CI)
	No			Yes				
	Event	PY	Rate^#^	Event	PY	Rate^#^		
All	2007	397402	5.05	780	99287	7.86	1.56 (1.43, 1.69)***	1.38 (1.27, 1.50)***
Gender								
Women	1640	314062	5.22	617	78532	7.86	1.51 (1.37, 1.65)***	1.34 (1.22, 1.48)***
Men	367	83340	4.40	163	20755	7.85	1.78 (1.48, 2.15)***	1.57 (1.30, 1.90)***
Stratify age								
≤ 40	523	195115	2.68	247	49274	5.01	1.87 (1.61, 2.17)***	1.53 (1.30, 1.78)***
>40	1484	202287	7.34	533	50014	10.7	1.45 (1.32, 1.61)***	1.29 (1.17, 1.43)***
Comorbidity^‡^								
No	984	283930	3.47	261	54508	4.79	1.38 (1.20, 1.58)***	1.41 (1.23, 1.61)***
Yes	1023	113472	9.02	519	44779	11.6	1.29 (1.16, 1.43)***	1.35 (1.21, 1.50)***
Vertigo								
No	1730	381760	4.53	642	93096	6.90	1.52 (1.39, 1.67)***	1.38 (1.26, 1.52)***
Yes	277	15642	17.7	138	6191	22.3	1.26 (1.03, 1.55)*	1.25 (1.01, 1.54)*
Insomnia								
No	1109	292982	3.79	328	59390	5.52	1.46 (1.29, 1.65)***	1.39 (1.23, 1.57)***
Yes	898	104419	8.60	452	39896	11.3	1.32 (1.18, 1.48)***	1.32 (1.18, 1.48)***
Anxiety								
No	1847	380827	4.85	617	87620	7.04	1.45 (1.33, 1.59)***	1.34 (1.22, 1.46)***
Yes	160	16575	9.65	163	11667	14.0	1.47 (1.18, 1.83)***	1.57 (1.26, 1.95)***
Hearing loss								
No	1927	395100	4.88	729	98279	7.42	1.52 (1.40, 1.66)***	1.36 (1.25, 1.48)***
Yes	80	2302	34.8	51	1009	50.6	1.48 (1.04, 2.10)*	1.42 (0.99, 2.03)
Follow-up time^‡^								
≤3 years	801	146730	5.46	387	36588	10.6	1.93 (1.71, 2.18)***	1.69 (1.50, 1.91)***
>3 years	1206	229154	5.26	393	57259	6.86	1.30 (1.16, 1.46)***	1.18 (1.05, 1.33)**

PY, person-years.

Rate^#^, incidence rate, per 1000 person-years; Crude HR*, relative hazard ratio; Adjusted HR^†^: multivariable analysis including age, gender, urbanization level and comorbidities of vertigo, insomnia, anxiety, and hearing loss; *p < 0.05, **p < 0.01, ***p < 0.001.

Comorbidities^‡^: Patients with any one of the comorbidities vertigo, insomnia, anxiety, and hearing loss were classified as the comorbidity group.

^‡^The follow-up time is partitioned into 2 segments (years ≤3, and >3 years) by first quartile.

### Risk of Tinnitus in Hyperthyroid Patients

After adjusting for age, gender and comorbidities of vertigo, insomnia, anxiety, and hearing loss, hyperthyroidism patients had a 1.38-fold risk of tinnitus (95% CI = 1.27-1.50) compared to non-hyperthyroidism controls. When stratified by sex, both the sex-specific hyperthyroidism cohort and non-hyperthyroidism cohort had a significant relative risk of tinnitus in women (adjusted HR=1.34, 95% CI=1.22-1.48) and men (adjusted HR=1.57, 95% CI=1.30-1.90). The incidence of tinnitus increased with age in both cohorts, however, the relative risk of tinnitus in the age-specific hyperthyroidism cohort compared to the non-hyperthyroidism cohort was higher in the younger group (adjusted HR = 1.53; 95% CI = 1.30–1.78).

### Tinnitus and Comorbidities

The relative risk of tinnitus in the comorbidity-specific hyperthyroidism cohort compared to the non-hyperthyroidism cohort was significant for patients presenting with any comorbidity (adjusted HR = 1.35; 95% CI = 1.21-1.50), and for patients with no comorbidity the relative risk of tinnitus was also significantly higher in hyperthyroidism cohort compared to the non-hyperthyroidism cohort (adjusted HR = 1.41; 95% CI = 1.23–1.61). Sub-group analysis in follow-up time showed that the higher risk of tinnitus occurred during the first 3 years of the follow-up period (adjusted HR = 1.69, 95% CI = 1.50-1.91), and was reduced with increasingly longer periods to 1.18 (95% CI = 1.05-1.33) after the first 3 years of follow-up.

### Risk of Tinnitus in Hyperthyroidism by Cox Proportional Hazard Analysis

Results of univariable and multivariable Cox proportional hazard analyses for detecting associations between tinnitus and hyperthyroidism are shown in **Table 3**. The adjusted HR of developing tinnitus was a 1.38-fold (95% CI = 1.27-1.50) increased risk for patients with hyperthyroidism than those without hyperthyroidism. Women had a 1.10-fold (95% CI = 1.00-1.21) increased risk of developing tinnitus compared with that for men. The adjusted HR of developing tinnitus was increased 1.02-fold (95% CI = 1.01-1.02) with age. The risk of developing tinnitus was higher for patients with comorbid vertigo (adjusted HR = 2.04, 95% CI = 1.82-2.29), insomnia (adjusted HR = 1.58, 95% CI = 1.45-1.72), anxiety (adjusted HR = 1.19, 95% CI = 1.05-1.34), and hearing loss (adjusted HR = 4.17, 95% CI = 3.48-4.99).

**Table 3:** Associations between tinnitus, hyperthyroidism and covariates: Cox model with hazard ratios and 95% confidence intervals.

Variable	Crude		Adjusted^†^	
	HR	(95% CI)	HR	(95% CI)
Hyperthyroidism				
Yes	1.56	(1.43, 1.69)***	1.38	(1.27, 1.50)***
No	1	(reference)	1	(reference)
Gender				
Women	1.13	(1.03, 1.24)*	1.10	(1.00, 1.21)*
Men	1	(reference)	1	(reference)
Age, years	1.03	(1.03, 1.03)***	1.02	(1.02, 1.02)***
Urbanization level				
1 (highest)	1	(reference)	1	(reference)
2	1.14	(1.04, 1.26)**	1.10	(1.00, 1.22)*
3	1.06	(0.95, 1.19)	1.02	(0.91, 1.14)
4 (lowest)	1.25	(1.12, 1.38)***	1.04	(0.94, 1.16)
Baseline comorbidities				
Vertigo				
No	1	(reference)	1	(reference)
Yes	3.82	(3.44, 4.24)***	2.04	(1.82, 2.29)***
Insomnia				
No	1	(reference)	1	(reference)
Yes	2.32	(2.15, 2.50)***	1.58	(1.45, 1.72)***
Anxiety				
No	1	(reference)	1	(reference)
Yes	2.18	(1.94, 2.45)***	1.19	(1.05, 1.34)**
Hearing loss				
No	1	(reference)	1	(reference)
Yes	7.36	(6.17, 8.77)***	4.17	(3.48, 4.99)***

Crude HR*, relative hazard ratio; Adjusted HR^†^: multivariable analysis including age, gender, urbanization level and comorbidities of vertigo, insomnia, anxiety, and hearing loss;

*p<0.05, **p < 0.01, ***p < 0.001.

## Discussion

This is the first longitudinal cohort study to demonstrate a positive association between hyperthyroidism and tinnitus, as well as an increased risk of developing tinnitus in hyperthyroid patients. Results have shown that, relative to individuals without hyperthyroidism, patients with diagnosed hyperthyroidism was more prone to develop tinnitus. Our study found higher risk of tinnitus occurred during the first 3 years of the follow-up period, and that the cumulative incidence of tinnitus was still higher in the hyperthyroidism cohort than that in the non-hyperthyroidism cohort during up to 12 years of follow-up. Additionally, patients with hyperthyroidism who had comorbid vertigo, insomnia, anxiety and/or hearing loss were at significantly higher risk of developing tinnitus than those without these comorbidities.

Previous studies have reported a positive association between tinnitus and thyroid disease history (2). As in the present study, associations with comorbidities were also noted, including gender, smoking, stress, sleep, hearing loss, hyperlipidemia, osteoarthritis, rheumatoid arthritis, asthma, and depression (2). Indirect evidence of the relationship between hyperthyroidism and tinnitus may lie in the link between hyperthyroidism and hearing loss. A statistically significant higher hearing threshold was found among patients with Grave’ s disease than that in the normal population, including significant hearing loss at high frequencies in the hyperthyroidism group (12).

Gender differences have been reported repeatedly in previous studies, including Basso et al. (13), who identified gender-specific risk factors, stating that women with bothersome tinnitus had higher rates of cardiovascular disease and thyroid disease; anxiety was also associated with tinnitus and levels were higher in women than in men. Our study also found a significantly higher incidence of tinnitus in hyperthyroid women than in hyperthyroid men, with women having a 1.10-fold increased risk of developing tinnitus than men. However, a study of older adult patients with persistent tinnitus found no significant differences between genders; the most prominent association with tinnitus was hearing impairment, regardless of age or gender (14). A systematic review of tinnitus-related studies found inconsistent results for gender between the included studies, with half of studies noting higher prevalence of tinnitus severity in males and the other half in females, although the prevalence of tinnitus itself was most often higher in males when age was more widely distributed (4). Study samples with older adults, however, mostly reported higher prevalence among women, which is more in line with other reports.

In the present study, the incidence of tinnitus increased with age, comparable to results of a previous study examining differences in age at tinnitus onset (15). Earlier onset was found associated with less distressing tinnitus while onset at older ages was distressing from the very beginning. It was suggested that an age-related decline in brain plasticity may account for the age-dependent severity. However, in our present study, while stratifying subjects by age (≤ 40 years *vs.* > 40 years) or by follow-up time to tinnitus after hyperthyroidism diagnosis (≤ 3 years *vs.* > 3 years), hyperthyroid patients were found to develop tinnitus at an earlier age or within the 3-year follow-up time after hyperthyroidism diagnosis than non-hyperthyroidism patients. Results of a study by Kim et al., 2015, showed that the prevalence of tinnitus was 16.0%–20.5% in those 20 to 54 years old and increased sharply after age 55 years, continuing to increase by 30% after 70 years (2). In another cross-sectional study by McCormack et al., 2016, reported that prevalence of frequent tinnitus increased as age increased, with a peak at 14.3% after age 60 years (3). In a recent study, Kim et al. (16), reported that a low thyroid-stimulating hormone level was associated with 2.78-fold higher odds of annoying tinnitus, in subgroup analyses, this association was apparent only in the female subgroup. However, in their study only patients > 40 years were included for analysis.

While associations between thyroid function and tinnitus have been suggested, the neuropathologic mechanism of tinnitus resulting from thyrotoxicosis has not been elucidated in detail. Whether it is a direct result of neurologic modulation by thyroid hormone or synergistic effects of catecholamine on auditory or non-auditory pathways still needs to be investigated. For example, establishing an animal model of thyrotoxicosis and tinnitus may help to further investigate the role of thyroid hormone in the development of tinnitus. In recent decades, however, the view of the tinnitus mechanism has focused more on centrally mediated consequences, that is, central gain followed by decreased sensory input may induce tinnitus (17–19).

Thyroid hormone has long been known to increase adrenaline sensitivity (20, 21). Hyperthyroid status produces variable changes in the number of adrenergic receptors or an affinity for catecholamines in different tissue, which may affect physiological function (22, 23). The synergistic effect of norepinephrine and thyroid hormone has also been suggested (24). Reduced vascular contractility has been observed in hyperthyroid rats (25). Further examination in humans demonstrated that thyroid hormone may influence peripheral blood flow (26).

Evidence from previous experimental studies reveal close interaction between the autonomic system, cochlear blood flow and cochlear function. Spoendlin and Lichtensteiger (1967) first demonstrated the presence of adrenergic fibers in the inner ear and described the direct influence of the sympathetic nervous system on cochlear receptor *via* the independent vascular system (27). Hozawa and Kimura used immunohistochemical examination to demonstrate the adrenergic neurotransmitter in the inner ears (28). Another early study observed that abnormal endolymph production may lead to cochlear dysfunction and autonomic imbalance will result in inner ear disorders (29). The concentration of catecholamines in the cochlea was found to change dramatically after superior cervical ganglionectomy (30). Cochlear blood flow also decreased by stimulating the sympathetic stellate ganglion in guinea pigs (31), as well as after cervical sympathetic stimulation (32). Further examination revealed an association between sympathetic nerves in the cochlea and the diameter of the feeding vessel (33), suggesting that cochlear blood flow is controlled by the sympathetic nervous system. The sympathetic fibers in the cerebral cortex cause vessel constriction and may ultimately influence cochlear, vestibular nuclei and cortex functions (34). Therefore, taken together, we suggest that cochlear pathology may result from autonomic imbalance related to excessive thyroid hormone and sympathetic over-activity. Central gain followed by reduced sensory input may then induce tinnitus. However, the neuropathological mechanism of tinnitus resulting from thyrotoxicosis remains to be examined in future experimental studies.

It is known from animal studies that thyroid hormone increases neuronal excitability in the brain, suggesting that not only hypothyroid insufficiency but thyrotoxicosis may produce adverse effects (35). Meanwhile, it is interesting that tinnitus and psychiatric conditions may share overlapped modulating pathways based on the role of the limbic system. Thyroid hormone receptor is widely distributed in limbic structures of the brain, and hyperthyroidism is associated with psychiatric disorders, which may arise from action of thyroid hormone on the limbic system (36–40). The influence of non-auditory pathways on tinnitus, such as the influence of the limbic system, has been addressed but without definitive results (41, 42).

### Strengths and Limitations

The main strength of this study was the longitudinal cohort design, and that we used the LHID2000. Data from LHID were physician clinical diagnosis instead of self-reported by patients. Use of the well-established LHID dataset which is a subset of the Taiwan NHIRD reduced the risk of sampling bias and reflected the real-world data. The long follow-up period and large sample size also provided considerable statistical power.

Nevertheless, this study has a few limitations. First, the LHID does not provide all pertinent variables, including laboratory findings such as thyroid-stimulating hormone levels, therefore patient inclusion was based on ICD-9 diagnosis code alone, no biochemical hormone levels were evaluated to confirm the diagnosis, patients with subclinical hyperthyroidism may have been included. Second, we did not analyze the effect of medication for thyroid dysfunction or comorbidities and tinnitus in the present study. It would be interesting to note if there were any differences in development of tinnitus in hyperthyroidism patients who were treated *versus* those not treated for comorbidities. Further study is warranted to study the effect of medication as well as to investigate the variables associated with risk of tinnitus in patients with confirmed hyperthyroidism diagnosis using hospital-based database.

### Conclusion

The incidence of tinnitus is associated with hyperthyroidism. Hyperthyroidism itself may be a risk factor for developing tinnitus. Clinicians should be aware of tinnitus development in patients with hyperthyroidism, particularly during the initial 3 years after hyperthyroidism diagnosis. The role of thyroid hormone in tinnitus, and other possible neuropathological mechanisms, require further investigation.

## Data Availability Statement

The original contributions presented in the study are included in the article/supplementary material. Further inquiries can be directed to the corresponding author.

## Ethics Statement

The studies involving human participants were reviewed and approved by Institutional Review Board of China Medical University. Written informed consent for participation was not required for this study in accordance with the national legislation and the institutional requirements.

## Author Contributions

T-YC had full access to all the data in the study and takes responsibility for the integrity of the data and the accuracy of the data analysis. Concept and design: T-CW. Acquisition of data: T-CW, C-JC, P-CC, and C-LL. Analysis and interpretation of data: All authors. Drafting of the manuscript: T-CW, C-JC, P-CC, T-YC, RT, and C-LL. Critical revision of the manuscript: T-CW, C-JC, P-CC, T-YC, RT, E-RR, CC, PM, C-DL, and M-HT. Statistical analysis: T-CW, C-JC, P-CC, T-YC, RT, and C-LL. Definition of intellectual content: T-CW, C-JC, P-CC, T-YC, RT, E-RR, CC, and PM. Clinical and experimental studies: T-CW, C-JC, and P-CC.

Administrative, technical, or material support: T-YC, C-DL, and M-HT. Obtaining Funding: T-CW, C-DL, and M-HT. Supervision: T-YC and RT. All authors contributed to the article and approved the submitted version.

## Funding

This work was supported by grants from the China Medical University in Taiwan (CMU107-N-30). This study is supported in part by Taiwan Ministry of Health and Welfare Clinical Trial Center (MOHW109-TDU-B-212-114004) and China Medical University Hospital (DMR-110-222), Tseng-Lien Lin Foundation, Taichung, Taiwan.

## Conflict of Interest

The authors declare that the research was conducted in the absence of any commercial or financial relationships that could be construed as a potential conflict of interest.

## Publisher’s Note

All claims expressed in this article are solely those of the authors and do not necessarily represent those of their affiliated organizations, or those of the publisher, the editors and the reviewers. Any product that may be evaluated in this article, or claim that may be made by its manufacturer, is not guaranteed or endorsed by the publisher.
